# Exploitation, Criminalization, and Pecuniary Trade in the Organs of Living People

**DOI:** 10.1007/s11673-021-10091-6

**Published:** 2021-02-22

**Authors:** Hugh V. McLachlan

**Affiliations:** Glasgow Caledonian University, Cowcaddens Road, Glasgow, G4 0BA UK

**Keywords:** Organ sales, Exploitation, Altruism, Altruistic donation, Justice

## Abstract

It is often maintained that, since the buying and selling of organs—particularly the kidneys—of living people supposedly constitutes exploitation of the living vendors while the so-called “altruistic” donation of them does not, the former, unlike the latter, should be a crime. This paper challenges and rejects this view. A novel account of exploitation, influenced by but different from those of Zwolinski and Wertheimer and of Wilkinson, is developed. Exploitation is seen as a sort of injustice. A distinction is made between justice and fairness. To exploit someone is to take advantage of him or her unjustly. Exploitation pertains to the nature of actions, interactions, and transaction rather than to their outcomes or to how they are perceived by exploitees. Desperation on the part of one or other of the parties to a transaction does not preclude the giving of valid consent to the transaction. Disparities of power or wealth between the parties to a transaction do not indicate or entail that the transaction will be exploitative. A disparity in the benefits that arise from a transaction between the parties does not indicate or entail that exploitation has taken place.

## Introduction

In the United Kingdom and in most other countries, it is a crime to buy and sell organs of living human beings but not to donate them “altruistically” (Greasley 2014; Koplin 2018; McLachlan 1998, 1999; Saunders 2018). The organ in question is usually a kidney since we naturally have two but can survive with one. A common suggested justification for this difference in treatment is the assertion that the former arrangement, unlike the latter one, entails the exploitation of the organ providers. Exploitation is not in itself a crime. It does not follow that if and when the selling of the organs of living human beings is exploitative, it should be illegal. However, I shall argue that the purchase of an organ of a living human being is inherently no more exploitative for the seller than for the buyer and inherently exploitative for neither of them. I shall base my case on an analysis of “exploitation” that is influenced by but differs in significant respects from that of Zwolinski and Wertheimer and that of Wilkinson (Wertheimer 1996; Wilkinson 2003a, 2003b; McLachlan and Swales 2001; Zwolinski and Wertheimer 2016).

## What is Exploitation?

### Exploitation as Unjust Advantage Taking

The term “exploitation” is sometimes used in a morally neutral way as, say, when we talk about exploiting our resources or talents when we mean taking advantage of them. In the ethical sense of the term, exploitation is the taking advantage of people. However, to take advantage of someone is not always exploitation. It is wrong to treat other people in all respects merely as means to our own ends. It is not always wrong to use them in some respects merely to our own advantage. For instance, if we ask a stranger to give us directions, we might be said to be using him or her merely as a means to our own ends. We are taking advantage of the person but not thereby exploiting him or her. Similarly, when we buy food from a baker or, say, pay a dentist or a lawyer to provide a service for us, we are taking advantage of them and they are taking advantage of us. However, it does not follow that such relationships and transactions are intrinsically exploitative. Similarly, to take advantage of what might be unfair or unjust circumstances is not exploitation (Lawlor 2014; McLachlan and Swales 2001. For instance, a dentist, a divorce lawyer, and a criminal lawyer might take advantage of our toothache, of our marital discord, or of a false accusation of criminality against us to earn money from us without exploiting us no matter how unfair or unjust is the physical or mental anguish which occasions us to purchase his or her services.

To exploit someone is to take advantage of him or her wrongfully. Different instances of wrongful advantage taking might be wrong for different reasons. However, I suggest that exploitation should be defined specifically as unjust advantage taking. I suggest too that justice should be considered with reference to moral rights and that moral rights should be considered in relation to moral duties. Moral injustice is action that is in breach of a moral duty that corresponds to someone’s moral right as, for instance, when the moral duty of a promisor to keep a promise corresponds to the promisee’s right that the promise is kept or, say, the moral duty of a neighbour to return a pair of borrowed garden shears corresponds to the owner’s moral right that they are returned. I am offering a stipulative redefinition of the term “exploitation.” Hence, the question of the truth or correctness of my proposed definition of exploitation as unjust advantage taking does not arise. The relevant consideration is its potential usefulness. Does it lead to clarification or obfuscation? Does it illuminate significant distinctions that might otherwise remain hidden? Such usefulness or uselessness can be gauged only by using the term “exploitation” in the proposed modified way and seeing what happens.

Agencies as well as agents can have moral rights and moral duties. They can exploit and be exploited. For instance, tax evasion and the fiddling of social security benefits are, if and when they occur, both forms of exploitation (McLachlan 2005; McLachlan 2010).

For me, moral duties are the fonts of moral rights rather than vice versa (McLachlan 2010). We have, for instance, a moral duty not to coerce people. The moral right not to be coerced corresponds to and is derived from that moral duty, in my view. Coercion, when it occurs, derives from the nature of the action of the person who is coercive rather than in the perception of that action by the person who is coerced or on the effect of the coercion on that person. Similarly, the validity or invalidity of consent in this context derives from the nature of the offering of money for the live organ rather than on the effect of the offer on the person to whom it is made. Offers can be coercive. Consent to them can be invalid as, for instance, when they are deceitful or deliberately misleading. However, my argument is not that trade in live organs in never coercive and that consent to it is always valid but that such trade is not inherently exploitative. Offers are not necessarily coercive or, on other grounds, wrong but they can be made wrongfully and coercively as, say, when women receive unbidden and unwelcomed offers of money for sex when they are walking alone in public places. The making of such offers can be harmful and wrongfully harmful. Similarly, the direct, uninvited offer of money for one’s kidney could wrongfully cause some people distress and psychological harm in some circumstances.

When there is an established legalized market for the buying and selling of any goods or services, the likelihood of the making or receiving of such harmful, distressing, wrongful, and coercive offers decreases, often to vanishing points. People can advertise their willingness to buy or sell the goods and services. They can choose to peruse the advertised offers of payment without being directly accosted by a prospective customer.

Consider some examples of how my notion of exploitation as unjust advantage taking would apply. To threaten someone with a gun and thereby steal, say, his watch is exploitation. To offer to buy a watch from someone is not necessarily exploitative. To force someone, say, to have sex regardless of his or her consent is exploitation. To offer payment to someone to have sex is not necessarily exploitative. To coerce or threaten someone is to contrive to make the circumstances of the exploited person appear less attractive to him or her if he or she were to refuse to do what you demand him or her to do. To offer money is to try to make the circumstances of the person to whom it is offered appear to be more attractive to him or her if he or she agrees to do what you request him or her to do. We are morally obliged not to coerce people. We are not similarly morally obliged to refrain from making attractive offers to them. Threats are—but offers are not—intrinsically wrongful. One can reject an offer without being harmed and without being wronged by the person who offers it. Even if one is not harmed by a coercive threat, one was wronged by the making of it. People are treated as objects, as means to the ends of those who threaten and coerce. Offers, on the other hand, can be made and accepted by people who treat each other as such.

Although offers are not in themselves coercive, wrongful, or harmful, people can, indirectly, be distressed and even psychologically harmed by their reactions and responses to them. Buyers and sellers can be remorseful to various extents. Their hopes and expectations are not always fulfilled. Those who refrain from undertaking particular transactions can also experience remorse. We can, for instance, bitterly regret having rejected, out of timidity, perhaps, or misplaced pessimism, some particular offers just as we can come to regret having accepted other ones. For instance, one can bitterly regret and be psychologically damaged by the aftermath of the acceptance or rejection of a proposal of marriage. However, it does not follow that such proposals are coercive or exploitative. We have a moral right not to be wrongfully psychologically damaged, which corresponds to the moral duty of other people not to cause psychological damage wrongfully. We do not have a moral right not to suffer psychological damage.

Suppose that a poverty-stricken parent realized that she could raise money that could improve the circumstances of her children by selling one of her kidneys but was terrified by the prospect of doing so and suffered from a feeling of guilt and consequently damaging distress. The availability of the offer, it could be said, worsened her own circumstances. Perhaps, in the circumstance, it was appropriate for her to feel moral guilt. On the other hand, her distress and feeling of guilt might have been completely misplaced. Either way, it does not follow that the availability of the offer was coercive, exploitative, or wrongful.

To acquire someone’s watch by threatening to shoot him if he does not give you his watch is to take advantage of him unjustly. The person has a moral right not to be so threatened. The thief has a moral duty not to threaten people with a gun. However, we are not under a moral obligation to refrain from offering other people money for their watches. People who have watches do not have a moral right not to be offered money for them. We do not have a moral duty to refrain from accepting watches that are offered to us in return for money. Similarly, while in most circumstances it would be insulting and thereby the breach of a (comparatively weak) moral duty gratuitously to ask people if they wanted to have sex with us in return for money, it would not be so in all circumstances. More importantly, it would not be a breach of a moral duty and the infringement of a corresponding right of the prostitute to accept such an offer of sex in return for money. Prostitution might or might not be immoral in many respects and a breach of moral duties and an infringement of moral rights on the part of one or both of the parties involved. However, the transaction is not inherently exploitative.

It might be said—whether or not truly—that the owner of the watch and the prostitute would offer the watch and the sex for sale only if and because they were desperate. However, even if that were the case, it would not render all subsequent or consequent transactions exploitative. We have a moral right not to be coerced by fellow human beings. We do not have a moral right not to be “coerced” by desperate circumstances. It is bad lack rather than injustice if circumstances rather than agents or agencies thwart or propel us.

Exploitation pertains to the nature of actions, interactions, and transaction rather than to their outcomes or to how they are perceived by exploitees. To deny that particular actions, interactions, and transactions are exploitative is not to claim that they are morally faultless. They might have some other moral defects. “Exploitative” and “morally wrong” are not synonymous terms.

### Zwolinski and Wertheimer and Exploitation

Writing in the *Stanford Encyclopedia of Philosophy*, Zwolinski and Wertheimer argue that:

To exploit someone is to take unfair advantage of them. It is to use another person’s vulnerability for one’s own benefit. Of course, benefitting from another’s vulnerability is not always morally wrong—we do not condemn a chess player for exploiting a weakness in his opponent’s defence, for instance. But some forms of advantage-taking do seem to be clearly wrong, and it is this *normative* sense of exploitation that is of primary interest to moral and political philosophers. (Zwolinski and Wertheimer 2016)

Although something can both unfair and unjust, to exploit someone is to take an unjust advantage of him or her, in my view. Furthermore, I do not agree that exploitation necessarily involves playing on a vulnerability or weakness on the part of the exploited person.

Zwolinski and Wertheimer contend that exploitation can be considered in two different ways: in terms of the consequences or in terms of the nature of the behaviour in question. They write:

First, it may refer to some dimension of the *outcome* of the exploitative act or transaction, that is, the transaction is substantively unfair … Second, to say that *A* takes unfair advantage of *B* may imply that there is some sort of defect in the *process* by which the unfair outcome has come about, for example, that *A* has coerced *B* or defrauded *B* or has manipulated *B*. (Zwolinski and Wertheimer 2016)

Wilkinson similarly writes that: “Behind the moral sense of ‘exploitation’ lie two distinct worries: one about *disparity of value*, the other about wrongful use. These can usefully be thought of as two different kinds of exploitation” (Wilkinson 2003a, 26).

I think that exploitation relates in Zwolinski and Wertheimer’s terms solely to the nature of the process, not the outcome, and in Wilkinson’s terms solely to the wrongful use and not to the disparity of value.

### Exploitation, Justice, and Fairness

I suspect that the term “fairness” is, like the terms “beauty” and “humour,” indefinable. We can give examples of particular things which we consider to be fair, humorous, or beautiful, but that is not the same as giving a linguistic definition of the words “fair,” “humorous,” or “beautiful.” Reasonable people can reasonably disagree over what is fair in particular circumstances. Different people can reasonably consider different things to be fair. Furthermore, the same person can consider the same thing to be fair or unfair depending on the point of view it is considered from. Moreover, to treat one particular person fairly might involve treating others unfairly (McLachlan 2005, 28–38). We can change our mind about what is beautiful by, for instance, becoming aware of the judgements that other people make and the reasons they suggest for making them. We can similarly change our minds about what we consider to be fair.

Since Rawls is particularly noted for his account of justice as fairness, it is useful to indicate here how my views relate to his. Rawls typically appeals to intuitions rather than definitions in his writings on justice and fairness. He presents what he calls a “political conception of justice.” This consists of principles which, Rawls argues, would be accepted by rational egoists in particular specified circumstances who value freedom and equality because they would intuitively grasp their fairness. “Justice” thus considered is, for Rawls, a subcategory of “fairness,” which remains undefined. He writes:

Let’s now survey briefly some of the basic ideas that make up justice as fairness in order to show that these ideas belong to a political conception of justice . . . [T]he overarching fundamental intuitive idea, within which other basic intuitive ideas are systematically connected, is that of society as a fair system of cooperation between free and equal persons. Justice as fairness starts from this idea as one of the basic intuitive ideas which we take to be implicit in the public culture of a democratic society. (Rawls 1985, 231)

While Rawls considers justice with regard to principles of social cooperation, my conception of justice relates to social interactions and transactions. I think that although they can overlap, justice and fairness are different. Justice and fairness can clash. One is not a subcategory of the other. However, while I suggest a definition of justice, with regard to its form rather than its substance, I have no specific definition of fairness to offer. Like Rawls, I appeal to our intuitions. When we say that something is fair, we are, I suggest, appealing to our moral intuition of the reasonableness and moral appropriateness of particular actions and arrangements of things. Our intuitions of fairness are, I think, often similar, although they can differ just as our own notions of fairness can change. Not only can they change but, I suggest, our judgements about the fairness or unfairness of particular things can be developed, amended, and refined.

Imagine that a group of people is standing in a circle at the centre of which is the sculpture of a horse. Is the artefact beautiful? We need not claim that there is a correct unequivocal answer to that question even if, as I do, one rejects the tempting subjectivist notion that beauty is in the eye of the beholder (Hume 1910). From different vantage points in the circle, the object might correctly and incorrectly be said to be beautiful. It depends on where one looks at it from. So, by analogy, it is with fairness. Consider, for instance, the biblical parable of the labourers in the vineyard. Many people argue it is fair that people who do the same job equally well should be paid the same by their employers for doing so and that people who do more or better work should be paid more. In Matthew’s gospel, chapter 20, verses 1–16, the parable of the labourers in the vineyard illustrates some of the complexities involved with this application of the idea of fairness. We cannot simply say that fairness means equality, since things that are the same in one respect are often different in another respect. We cannot use the idea of equality to tell us what respects of sameness should count and which should be disregarded.

The owner of a vineyard hired labourers to work for a day for an agreed fee. Later in the day, at different times, he hired other labourers to work for the remainder of the day. When they were paid, all were paid the same, no matter how long they had worked. There were understandable complaints from those who worked longer. Were they treated unfairly? It depends upon how one looks at the matter. Whether or not they were all treated fairly, it might be said that they were all treated justly if each received from the owner no less than he had promised to pay them and they had worked for no longer than they had offered to work for the agreed payment. He had a moral duty to treat them all justly. Justice in the circumstances need not imply impartial treatment as it would, say, in the relationship between a teacher and a class of students in the marking of their exams or coursework. It is not obvious that he had a moral duty to treat them all fairly in all respects. To treat some of them more generously than was required by mere justice and mere fairness might have been morally permissible however unfair this appeared—and however unfair this was—to some of the labourers.

Some things are both unfair and unjust. However, fairness and justice are not the same thing. Consider a criminal trial for a serious offence such as rape. Someone who is tried for such an offence has a moral right to a fair trial and to be found guilty only if his or her guilt is established beyond all reasonable doubt by the evidence presented during the trial. If a trial is a fair and a just one in which members of the jury and the agents and agencies of the state and legal process fulfil their moral duties, neither the individuals nor the agencies act wrongfully or exploitatively. Nonetheless, the process and the outcome of the trial might well be rightly considered to be very unfair to one or more of the people involved, particularly to the alleged victim if, say, the alleged rapist actually did commit the crime for which there was insufficient evidence to establish his guilt.

While Zwolinski and Wertheimer consider exploitation to be the taking of an unfair advantage, I prefer to confine exploitation to particular sorts of unjust actions. As we have seen, they say that there might be an instance of the exploitation of B by A if: “… for example … *A* has coerced *B* or defrauded *B* or has manipulated *B*.” I would consider them to be instances of exploitation only if the coercion, fraudulence, or manipulation were unjust and thereby constituted the breach of a moral duty on the part of A that was the infringement of a corresponding moral right of B. Mere unfairness would not be exploitation in my view. At least in general, we are morally obliged not to defraud or coerce people, and we have a corresponding moral right in general not to be defrauded. In general, fraud and coercion are not merely unfair, they are unjust.

However not all manipulation is unfair and not all unfair manipulation is unjust. For instance, spouses and partners frequently—typically one might say—have strong views about how the other should look and behave and often try to manipulate them to conform to their wishes. This can be done in ways that are immoral and illegal, but not all such manipulation is immoral and illegal. Not all such manipulation is exploitative. For instance, to refuse to invite one or other of one’s flirtatious friends to visit one’s home because of a fear he or she might seduce one’s spouse might or might not be unfair manipulation, but it is not unjust. Spouses are not under a moral duty to invite such feared rivals to their homes. It is not a moral right of spouses that such flirtatious people are given such invitations by their spouses. However, it would be a cruel, unjust sort of manipulation to try to deprive one’s spouse of friends in general by threatening to assault them or by telling them, untruthfully, that one’s spouse did not want to see them.

### Exploitation and Vulnerability

Imagine a public beach on which there lies a very large person. Suppose that someone decided to use that person’s body as a windbreak. Is this an exploitative interaction? It depends, in my view, on whether it involves a breach of a moral duty of the person who seeks shelter and the infringement of a corresponding right of the recumbent large person. For instance, if the person who sought the shelter of his or her body lay so close to the large person that it was an invasion of the person’s privacy and an encroachment of his or her space, this would constitute exploitation. It would clearly be an invasion of privacy if the person put his or her body against that of the large person or even lay beside the large person on his or her moderately sized towel. It would be exploitative behaviour even if the large person were unaware that he or she had been wrongfully used, even if, for instance, he or she had slept through the incident. The exploitation is not necessarily harmful, although it is wrongful, to the one who is exploited. Indeed, it would be exploitation even if the large person very much silently enjoyed and benefited from the warmth provided by the very close proximity of the uninvited shelter-seeker. On the other hand, if the shelter-seeker lay close enough to the large person to benefit from the protection from the wind provided by his or her body but not close enough to constitute an invasion of the person’s personal space, it would not be exploitation, in my view. To treat a person merely as a commodity or as an object in all respects is wrongful. However, it is not always wrongful to treat a person’s body as an object in some respects whether or not the person concerned consents or not. The shelter-seeker uses the bulk of the large person, but it would be strange to suggest that the size of the person’s body was necessarily a weakness or vulnerability. The person might be, say, an American footballer or an Olympic athlete. Exploiters can, like experts in judo, direct the strengths of other people to their own use.

Notice too that even if the large person were obese and his or her bulk were considered as a weakness, it is not necessarily exploitative to take advantage of it as a wind break. Again, it depends on whether or not it thereby breaches a moral duty of the shelter seeker not to encroach and the corresponding moral right of the recumbent person not to have someone lying too closely nearby.

Similarly, kidnappers can wrongfully use the love of wealthy parents for their children for their own ends. Wealth and love of one’s children are not normally thought of as weakness or symptoms of vulnerability. The holding of someone to ransom with the threat of death if money is not paid for his or her release would seem to be a clear example of exploitation although exploitation is not the only wrongful or the worst thing about the activity. For the sake of simplicity, let us imagine a situation where the person who kidnaps and detains the hostage also demands and collects the ransom. Let us assume that the hostage is a child and that the ransom provider is a parent of the child. It would seem clear that the hostage taker is an exploiter. The hostage is exploited. The exploiter takes wrongful advantage of him. He does so in a way that is a breach of his moral duty not to detain people against their wills and an infringement of the moral right of the hostage not to be so detained. There is too, one might suppose, a moral duty not to threaten to kill people and a corresponding right not to be so threatened.

However, it would seem plausible to say that the ransom provider is also exploited even although his or her intervention is voluntary, unlike that of the hostage. They are both exploited but the exploitation of the hostage is more heinous and more clear-cut than that of the ransom provider. There is not merely an interaction but a transaction between the hostage taker and the ransom provider, unlike the relationship between the hostage and the hostage taker. The ransom provider and the hostage are both wronged by the exploitative hostage taker although in different ways. The hostage taker manipulates the circumstances of the ransom provider to turn what would otherwise be a request that is easy to reject—“Give me £100,000!”—into a demand which is difficult and psychologically costly for the ransom provider to disobey. Although the ransom provider consents to the payment of a ransom, the consent is not of any moral significance. It is not valid consent because of the coercion applied by the hostage taker. Coercion as such does not invalidate consent. What matters here is that the ransom provider was coerced by the person to whom he consented to give the money. Coercion of B by A rules out the giving of valid consent by B to A. Coercion of B by C does not rule out the giving of valid consent by B to A. “Coercion” of B by circumstances does not rule out the giving of valid consent by B to A.

Wilkinson writes: “The most exploitable people are those who are *both* very useful and vulnerable. A young woman who was both sexually attractive and very poor would be an obvious example” (Wilkinson 2003a, 24). This takes a narrow view of the matter. It depends on, for instance, what sorts of powers people have and what sorts of strategies they are capable of devising and prepared to employ in the pursuit of what particular goals they choose to pursue. For instance, it is not because they could not afford to do so or because they do not want to have sex that most men do not consult with prostitutes. For various reasons they choose not to pay for their services even when the services are cheap—with regard to many men, one might say, especially if the services are cheap. Some of them might have resorted to the services of a prostitute they could not afford even if they spurn the identical services of those they could.

The weak can exploit the strong. The poor can exploit the rich. Such exploitation might be less shockingly morally heinous, less mean, and less uncharitable than the exploitation of the weak by the strong and of the poor by the rich, but it is, nonetheless, exploitation. For instance, a pick-pocket acts unjustly and exploits those he skilfully robs whether or not he is a rich or a poor pick-pocket and whether he steals from, say, a paratrooper or a choir boy or from, say, a merchant banker or a nurse. The desperation of poverty might induce some people to succumb to the temptation to exploit others, which they might have resisted had they, the latter, been better off. Those who have in abundance that which is coveted by other people can be more attractive targets than the poverty stricken for potential exploiters. For instance, late at night in towns and cities, poor pedestrians mug rich ones rather than vice versa.

Wilkinson refers to the circumstances of workers who are poor and starving and who agree to accept very low wages for long hours of arduous, dangerous work and of people who are in debt to violent criminals and, out of desperation, resort to loan sharks. He writes: “These do seem to be classic cases of exploitation and are structurally identical to the case of the prostitute who may be keen to take on sex work given her poverty and lack of preferable alternatives” (Wilkinson 2003a, 74). Such circumstances are no more “classic” than are cases of exploitation with a different distribution of power and money between the exploited and the exploiters. For instance, if, in order to acquire money, you intend to kidnap a child and hold him or her to ransom, it would be more rational to kidnap the child of rich parents rather than one whose parents are poor. If one is rich and powerful and has an inclination to exploit someone with a view to become even richer and more powerful, it is unlikely that one would choose to kidnap a child and hold him or her to ransom. Such a venture is too risky. The consequences and the likelihood of failure are, in general, too great and the likely gain too slight for those who are already rich and powerful to want to undertake such a venture. Sometimes, it is more likely that the exploiter will be richer and more powerful than the exploited. Sometimes, the very opposite is more likely. It depends on the sort of exploitation that is involved rather than the presence of exploitation as such.

### Exploitation, Outcomes, and Disparities of Value

All the benefits of a particular interaction or transaction might go to one or other of the parties without the interaction or transaction being exploitative. All the benefits of a particular transaction might, say, go to either a bookmaker or to a punter. It does not follow that the transaction was exploitative. If a tourist in the street asked a passer-by for the directions to Glasgow Central Station, we would not consider the interaction to be exploitative even if only the tourist derived any benefit from it and even although the passer- by did not consent to the instigation, by the tourist, of the interaction and uttered a reply only to terminate it. Similarly, we might recall the large person who was recumbent on a beach. One might lie near him in order to use his body as a windbreak without exploiting him even though he gains nothing from the relationship as long as one does not lie too near to him and thereby treat him unjustly by invading his privacy.

Consider the circumstances where, with the intention of reneging on the arrangement, a man promises to marry a naive young woman in the hope and expectation of consequent sexual favours that otherwise would not be extended to him. This would certainly be morally wrong, possibly heinously so. It would be exploitative. However, there would seem to be no reason to conclude that the distribution of the net benefits of the relationship—were they commensurable—tilted towards the man rather than the woman. She was wronged whether or not she was harmed. It might well turn out that the women enjoyed the affair even more than the man did. It might turn out that the satisfied but exploited woman was happy to jilt her exploitative fiancé, much to his unexpected disappointment. Suppose that he caught a virulent sexually transmitted disease from the woman and consequently died after a long and painful illness while she lived a long and happy life with a loved and loving child who was conceived during the relationship. Whatever the outcomes of their actions, the man and the woman might, perhaps, each have exploited the other.

If A and B transact voluntarily, A agrees to do something for or with regard to A, and B agrees to do something for or with regard to B. We might presume that both imagine that the transaction will benefit or otherwise please them in some way that equals or exceeds what they consider to be the cost or inconvenience of the transaction to themselves. However, there is no reason to suppose that there will be, or that justice requires that there be, any sort of equivalence between the benefit to each or the cost or inconvenience to each of the parties to the transaction. In any case, the different benefits that the parties to a transaction enjoy are not always commensurate. The same can be said about the disbenefits or harms. From a transaction with my dentist, I gain relief from pain and he gains a sum of money. How can such benefits be commeasured? Why should we want to commeasure them? I will lose a particular sum of money. He will lose the time it takes to give me the dental treatment. Even if such losses were commensurate, it is not obvious that there would be any point to or relevance of the mensuration.

Suppose that in each of two isolated villages in the Scottish Highlands, there is a pub that charges seventy-five pence more than the normal price for a pint of beer in Scotland. Suppose that in village A, there is only one pub because its owner has driven all of his other competitors away by burning down their premises and threatening them with violence. Suppose that in village B, there is a pub because an enterprising publican from Glasgow decided to move there when it came to his attention that village B did not have a pub. I would suggest that the villagers in A are, while the villagers in B are not, being exploited by the owner of their local pub when they buy a pint of beer even though they pay the same and whether or not they think that they are being exploited. The exploitation relates to the intrinsic nature of the interaction and transaction rather than to its outcome and the distribution of its benefits. The publican in A does not merely provide potential customers with an option they can choose, in their circumstances, to their potential benefit. He wrongfully alters their circumstances to their detriment by limiting their range of options.

Consider the following example that is discussed by Zwolinski and Wertheimer:

An unexpected blizzard hits an area and people rush to the hardware store to buy a shovel. The hardware store owner sees the opportunity to make an abnormal profit and raises the price of a shovel from $15 to $30. If *B* agrees to pay $30 for the shovel, because the shovel is worth more than $30 to *B* under the circumstances, then the transaction is advantageous to both parties. If *B* is exploited, it is because *B* has paid too much … We need not deny that *B* benefits from these transactions, all things considered. Rather, *A* may exploit *B* if *B* pays too high a price for what she gains or does not receive enough for what she gives.

A mutually advantageous transaction is arguably (wrongly) exploitative only if the outcome is (in some way) unfair to *B*. (Zwolinski and Wertheimer 2016)

I do not consider this to be exploitative even if the outcome might, in some respects, be considered to be unfair to B. To insist that B should not be disadvantaged by and that A should not profit from the blizzard is not fair to A. The crucial point, in my view, is whether or not A is in breach of a moral duty and B suffers the infringement of a corresponding right. One might consider the supposed fairness of the situation from various different perspectives. For instance, rather than say that it is unfair to B that he or she has to pay more for the shovel when there is a blizzard, why not say that it is unfair to A that he or she receives less money for the shovel when there is not a blizzard? Suppose that A had only one shovel left and that while B offered only the normal price of $15, C, another of A’s regular customers, was prepared to pay $30 for it. It seems to me that it would be perfectly reasonable for A to sell the shovel to C for $30. Whether or not it would be fair to B, it would not be in breach of a moral duty on the part of A nor the infringement of a moral right of B: it would not be unjust; it would not be exploitative. To refuse to accept the offer of $30 for the shovel might, very reasonably, be considered by C to be unfair.

It is one thing to say that it would be morally commendable to refrain from taking a particular advantage of someone and saying that it is exploitative—a breach of moral duty—to do so. If a football player kicks the ball out of play because he thinks that a member of the other side is slightly injured, that might be commendable, but it is not necessarily exploitation to refrain from doing so. Similarly, it might be uncharitable to charge more for a shovel than one could afford to accept but it is not exploitative. By the same token, it might be uncharitable to offer less for a shovel than one could afford to pay, but it is not exploitative. Why should we say that A should not take advantage of the unusually bad weather rather than that B should not take advantage of the generally better weather? Why, indeed, make either claim?

## Is the Buying and Selling of the Organs of Living People Inherently Exploitative?

Consider the reasons commonly given for suggesting that the buying and selling of the organs of living people is exploitive. According to Radcliffe-Richards et al.: “The commonest objection to kidney selling is expressed on behalf of the vendors: the exploited poor, who need to be protected against the greedy rich” (1998, 1950). They continue: “It is said that they are likely to be too uneducated to understand the risks, and that this precludes informed consent. It is also claimed that, since they are coerced by their economic circumstances, their consent cannot count as genuine” (1998, 1950).

Radcliffe-Richards and her colleagues defend such sales, unlike Greasley, who is a staunch opponent of them. According to her:

In a nutshell, the policy objection is that rich people ought not to be able to take advantage of their poorer neighbours to the point of buying them out of their organs. Invariably, it is claimed, any market in human organs will be structured with poor people on the supplying end and richer people on the receiving. Since rational people would only sell an organ in circumstances of severe economic desperation, it is suggested that the practice would amount to straightforward advantage taking of the organ vendors. (Greasley 2014, 52)

With regard to the issue of “defective consent,” she argues that:

… the real concern does not turn on consent as such, but rather, on the claim that consensual or not, the kind of trading entailed by a living donor market in organs will almost always play on the natural disadvantages of the poor. The situation of the kidney-seller … [is] deeply disconcerting, not because we are unsure of whether he consented, but because we are certain that he never would have consented but for his poverty … (Greasley 2014, 52)

### Advantage Taking and the Sale of Kidneys

The purchaser might be said to take advantage of the provider of the organ just as the seller of the organ might be said to take an advantage of the buyer. However, neither necessarily takes a wrongful advantage of the other. The transaction is not necessarily exploitative. It is not the breach of a person’s moral duty and the corresponding infringement of the moral right of another to buy or sell his organ such as a kidney. Nevertheless, the purchaser might exploit the seller of the organ by, for instance, taking the organ and failing to pay the agreed price. The seller might exploit the buyer by, say, failing to disclose that his kidney is not as healthy as he has led the buyer to believe.

### Disparities of Power and the Sale of Kidneys

There might be, but there might not be, a disparity of wealth and power between someone who needs and wants to buy a kidney and someone who wants to sell one of his kidneys. If there is such a disparity, it might be in favour of the potential buyer or of the potential seller. In any event, such disparities of power and wealth need not necessarily lead to exploitation either of the buyer or the seller. Both can take advantage of the services offered by the other person without taking an unjust advantage, without breaching a moral duty and thereby infringing a corresponding moral right. Whether or not the buyer is more or less rich or more or less powerful than the seller, there is no reason to suppose that the contemplated transaction will necessarily be exploitative. If the transaction turns out to be an exploitative one, there is no reason either a priori or prima facie to suppose that the exploiter will be the buyer rather than the seller.

Suppose that someone, person A, needs and wants to buy a kidney and that someone else, person B, needs or wants money and is prepared to sell one of his or her kidneys to get it. It would make sense for A to seek a poor healthy person who wants to sell a kidney rather than a rich one in order for a deal to be more readily affordable. Similarly, it would make sense for B to seek a rich rather than a poor person who wants to buy one of his kidneys in order to get a higher price for the sale. However, it does not follow that there need be a vast difference in the power or wealth of A relative to B or that A need be the more powerful or the wealthier party. In any case, “power” is not a single entity or a force of a uniform nature. There are powers of different sorts, not all of which are commensurate. Who was more powerful—President Kennedy or Lee Harvey Oswald? Who has more power—someone who can offer the chance of life to another person who might otherwise die or someone who can merely offer money to someone who does not face the prospect of imminent death? There is, perhaps, no straightforward unequivocal answer to these questions (McLachlan 1981). Furthermore, it need not matter whether there are large differences in power between A and B. B could exploit A in such a transaction concerning the sale of A’s kidney to B, and B could exploit A, but neither need do so: exploitation is not inherent to such a transaction. For instance, A and/or B might lie to the other person about, say, the circumstances of his or her health or wealth. A might receive the kidney and fail to pay for it or fail to pay as much as he agreed to pay. B might receive the payment yet fail to deliver the agreed kidney, even if he delivers a kidney.

### Disparities of Benefits and the Sale of Kidneys

Both A and B are seeking to gain from the transaction. They can both do so without treating the other party unjustly whether or not there is a disparity between the benefits that each received. After all, in some non-exploitative interactions, only one of the parties benefits from the interaction. If there is a disparity, it might be in favour of B as readily as in favour of A. Individual A might be better off with the acquisition of a kidney and the loss of £X. Individual B might be better off with the acquisition of £X and the loss of a kidney. It is not clear that there is a disparity in the benefit that each derives from the transaction. It is not clear that the benefit of each is commensurate. What is the greater benefit: the continuation of life with the prospective alternative of death or, say, an increase of wealth to or beyond the level of subsistence with the prospective alternative of, say, abject poverty for one’s self and one’s family? The correct answer to this question, if there is one, is irrelevant with regard to whether or not the transaction is exploitative. Both can take advantage of the transaction and of each other without taking advantage unjustly whether or not one of the parties benefits much more than the other. One of the parties might gain far more from the transaction than the other without exploiting the other.

### Desperation, Consent, and the Sale of Kidneys

A and B might both be desperate but there is no reason to suppose that the potential seller will be more desperate than the potential buyer. Desperation can have different causes and can take different forms, which might be incommensurate. Nonetheless, desperation and an absence of feasible alternatives does not preclude the giving of valid consent, that is, the sort of consent that is sufficient to give a license to do something that might have been otherwise morally prohibited. We can properly engage in transactions with desperate people as, for instance, the potential buyers or sellers of organs such as kidneys. Desperate circumstances—whether economic ones or not—cannot wrongfully coerce or exploit people: only agents and agencies can do so since only they can have moral duties the breach of particular ones of which constitutes exploitative behaviour. The buying and selling of the kidney of a living person need not involve taking an unjust advantage of the buyer or the seller; it need not involve the breach of a moral duty and the infringement of a corresponding moral right.

Is a surgeon justified in removing someone’s kidney if the person consents to its removal because he or she is desperate to receive money from subsequently selling it? Is someone who is desperate to receive a kidney justified in purchasing one from someone who consents to sell it only because he or she is desperate to receive the money from its sale? Even if the correct answer to the first question were “no,” the answer to the second question might be “yes.” However, the correct answer to both questions is “yes.” Is the person with the kidney for sale justified in selling it to the potential purchaser who consents to buy it only because he is desperate? The correct answer to this question is also “yes.”

We are all under a prima facie moral obligation to refrain from surgically removing the kidneys of all other people. That moral barrier remains—and such an action would be a morally and legally unauthorized assault or worse—unless and until specific reasons serve to remove it. The consent of a patient can serve to remove that moral barrier for a suitably qualified surgeon if the surgeon has good reason to believe that the consenting patient is sufficiently and appropriately informed about the likely medical outcomes of the operation and the possibility of unknown and unknowable ones. Whether or not the patient consented out of desperation is irrelevant to the validity of such consent in releasing the surgeon from the prima facie moral obligation in question.

We are all under a prima facie moral obligation to refrain from taking the property including the money of other people into our possession without permission to do so. Their consent constitutes such permission generally. However, when some offers something for sale, we have, prima facie, permission to take and assume we have acquired the property if we fulfil the conditions of the deal by paying for it. Consent can be assumed unless there are particular reasons for assuming that appropriate valid consent was not given. For instance, if the property were known by us to have been stolen, the consent of the current possessor would be invalid with regard to permitting us to acquire it. However, that the person is desperate to sell the object or service or, rather, desperate to receive what was given in return for selling it, does not invalidate the consent given or the permission to acquire that is granted by it.

Suppose that it was known by someone who needed and wanted to buy a kidney, person A, that a kidney had been removed from someone by a surgeon without the valid consent of the patient, person B. It does not follow that any consent subsequently given by B to A for the sale of the kidney is invalid. It does not follow that the purchase of B’s kidney by A would be exploitation—that is, wrongful, unjust advantage-taking by A that constituted the breach of a moral duty by A and the infringement of a corresponding moral right of B.

It is possible that those who want to sell one of their kidneys are insufficiently educated to understand the risks of getting one of their kidneys removed, but there is no reason for thinking that they must be so uneducated. That would affect the consent given to the surgeon for the removal of the kidney rather than to the purchaser for the sale of it. The consent is such as to waive the right of the vendor to keep the kidney. The consent is sufficient to license the purchaser to take the kidney. It is sufficient to ensure that the purchaser is a purchaser and not an exploiter and a thief. Similarly, a prospective buyer of a kidney might be unaware of the known risks of having an operation to receive a healthy kidney from a healthy living donor. That might affect the validity of the consent that he or she is able to give to a surgeon to perform such an operation, but it does not invalidate the consent that he or she can give to the seller of the organ to authorize the latter’s acceptance of money in return for the kidney.

Every day, in hospitals throughout the country, people are told that, if they do not have particular operations and medical treatments, they will die. It does not follow that they cannot give valid consent to the operations and treatment. We do not normally say that the patients cannot give valid consent to the procedures since the only alternative to consenting to them is death. Wilkinson quotes Zwolinski and Wertheimer with approval on this point:

If A (a physician) should say to B (a patient), “You can choose to have this leg amputated or you will die,” we don’t say that B’s decision to have his leg amputated is coerced because death is an unacceptable alternative. Rather, we seek B’s informed consent to the procedure. (Wilkinson 2003b, 177)

As Wilkinson comments: “It would be quite bizarre to rule out the possibility of valid consent here just because of the lack of acceptable alternatives” (Wilkinson 2003b, 177).

If desperation and the absence of acceptable alternatives do not rule out the possibility of giving valid consent in this instance, we have no reason to suppose desperation and the absence of acceptable alternatives will rule it out in all other instances. That one would not or could not have wanted to turn down an offer is not a factor that affects the giving of valid consent to its acceptance. It is an irrelevance. Otherwise we would be led to the ludicrous conclusion that we could give full valid consent only to those things that we do not feel strongly about. For instance, we could not give our valid consent to have sex with people we adore and whose charms seem irresistible but only with those whom we find relatively unattractive.

### The Sale of Kidneys and the Vulnerability of Donors

It is the stated position of Kidney Research UK that they support and encourage the “altruistic” donation of kidneys from living donors whether they are relatives, friends, or people who are unknown to or by them. However, they:

… do not condone any scheme whereby people receive a monetary reward in exchange for an organ. The decision to become a living organ donor is one which is extremely personal and should not be motivated, influenced or incentivised by the prospect of financial gain. (Kidney Research UK 2017)

Why not? Their answer is that:

Paid provision could very well result in the abuse and exploitation of vulnerable people—people who might have lost their jobs, be in severe debt or are under duress. The idea that you can sell one of your organs to pay off a substantial debt, such as a loan, would undoubtedly appeal to some people. However, if money is their only motivation, they might find that they come to regret such a decision at a later date. (Kidney Research UK 2017)

This is irrational. Paid provision might, but it need not, be exploitative just as altruistic donation might, but need not, be exploitative (McLachlan 1998, 1999). Donors too—not merely vendors—might come to regret their decision whatever their motivation was. For instance, they might become ill or lose the use of their remaining kidney regardless of whether they were pecuniary or non-pecuniary donors. If they are altruistic donating relatives, they might have more possible grounds for disappointment than pecuniary ones. For instance, they are more likely to be distressed if the person who receives the organ dies. They might be disappointed if other family members or the donor him or herself fails to express anticipated gratitude. They might be disappointed if the recipient of the organ proceeds to live a shameful life. They might be disappointed if, after donating a kidney to a particular relative, another one who they much prefer and who is a better person with more dependents comes to require a kidney transplant. They might be disappointed if, after giving away a kidney, they come in later life to have a dreadful economic problem which they could have solved by the sale of a kidney if only they had not given one away.

## Conclusion

There might—or might not be—be good arguments for suggesting that the pecuniary trade in the organs of living people, such as kidneys, should be illegal (Rippon 2014; Semrau 2015). However, that such transactions are inherently exploitative is not one of them. To criminalize the buying and selling of such organs on the basis of alleged exploitation while permitting and even applauding the unpaid donation of them is inconsistent.
